# Detection, Isolation, and Identification of Mycobacteria That Cause Nontuberculous Mycobacterial Disease and Tuberculosis

**DOI:** 10.3390/pathogens14121302

**Published:** 2025-12-18

**Authors:** Lyudmila Severova, Dmitrii Giller, Inga Enilenis, Patimat Gadzhieva, Galina Shcherbakova, Oleg Kesaev, Vadim Koroev, Olga Frolova, Anna Popova, Alexandr Ilyukhin, Valeria Basangova, Elena Belova, Elham Pahlevani Gazi, Irina Taushkanova, Ivan Martel

**Affiliations:** 1 Department of Phthisiopulmonology and Thoracic Surgery Named After M.I. Perelman, Sechenov First Moscow State Medical University, Trubetskaya St. Bldg. 8\2, 119435 Moscow, Russia; giller_d_b@staff.sechenov.ru (D.G.); enilenis_i_i@staff.sechenov.ru (I.E.); gadzhieva_p_g@staff.sechenov.ru (P.G.); shcherbakova_g_v@staff.sechenov.ru (G.S.); kesaev_o_sh@staff.sechenov.ru (O.K.); koroev_v_v@staff.sechenov.ru (V.K.); popova_a_a_2@staff.sechenov.ru (A.P.); ilyukhin_a_n@staff.sechenov.ru (A.I.); basangova_v_a@staff.sechenov.ru (V.B.); 2 Department of Phthisiopulmonology, Pirogov Russian National Research Medical University, 117997 Moscow, Russia; 3 Department of General Hygiene, F. Erismann Institute of Public Health, Sechenov First Moscow State Medical University, 119435 Moscow, Russia; belova_e_v@staff.sechenov.ru; 4 N.V. Sklifosovskiy Institute of Clinical Medicine, Sechenov First Moscow State Medical University, Trubetskaya St. Bldg. 8\2, 119435 Moscow, Russiataushkanova_i_a@student.sechenov.ru (I.T.)

**Keywords:** *nontuberculous mycobacteria* (NTM), *Mycobacterium tuberculosis complex* (MTBC), *nontuberculous mycobacterial* pulmonary disease (NTMPD), diagnosis

## Abstract

Pulmonary diseases caused by nontuberculous mycobacteria are increasingly becoming common worldwide and are occurring more frequently alongside pulmonary tuberculosis. Given that pulmonary diseases resulting from nontuberculous mycobacteria and pulmonary tuberculosis display similar features—such as clinical manifestations, imaging findings, and laboratory results—the accurate differentiation of each disease type is highly challenging. Mycobacterial culture, as a gold standard method, cannot be considered completely trustworthy because of low bacterioexcretion rates among nontuberculous mycobacterial pulmonary patients. Additional problems result from poor diagnosis. The treatment of lung diseases caused by nontuberculous mycobacteria is also difficult. This could be due to the wide spectrum of bacteria belonging to nontuberculous mycobacteria, as well as low bacterioexcretion. Therefore, bacterial sensitivity to drugs is insufficient. As a result, in this article, our intention is to explain the diagnostic difficulties of pulmonary diseases caused by *nontuberculous mycobacteria* and the *Mycobacterium tuberculosis complex*. The review seeks to outline promising directions for the development of novel diagnostic approaches in order to improve clinical decision-making and ultimately treatment outcomes.

## 1. Introduction

Clinically relevant mycobacteria are traditionally divided into three major groups: the *Mycobacterium tuberculosis complex* (MTBC), the causative agents of leprosy (*Mycobacterium leprae* and *Mycobacterium lepromatosis*), and the diverse group of nontuberculous mycobacteria (NTM) [1,2,3,4]. Although this division is not taxonomic, it remains of substantial clinical and epidemiological importance because each group differs in its pathogenicity, transmission patterns, diagnostic challenges, and treatment strategies. Leprosy, in contrast to MTBC and NTM infections, presents with distinct dermatological and neurological manifestations. Therefore, leprosy is generally not associated with diagnostic difficulties when differentiating it from nontuberculous mycobacterial pulmonary disease (NTMPD) or pulmonary tuberculosis (pTB) [1,2,3]. While rare, it is necessary to distinguish MTBC from leprosy-causing species and NTM, which includes more than 200 environmental species with variable clinical significance. Importantly, NTM infections are not subject to mandatory reporting in most settings, which limits the availability of reliable epidemiological data and creates challenges for effective prevention and control strategies [1]. It is therefore essential to differentiate *Mycobacterium tuberculosis complex* and nontuberculous mycobacteria, which cause NTMPD. Consequently, these are of interest to review.

NTM, which is ubiquitous in the environment and belongs to the *Mycobacterium* genus, can be found in different environments, such as soil, water, animals, and food [1]. NTM features are very different from MTBC. Examples include more sensitivity to acids and alkalines and more significant resistance to many antituberculosis drugs, which can lead to poor treatment effectiveness and, in turn, cause high financial burden and prolonged duration of low quality of life [2,3]. The prevalence of NTMPD is increasing globally [4,5,6].

NTM can affect both pulmonary and extrapulmonary tissues, but among them, NTMPD has a high prevalence. The rate of morbidity and mortality caused by NTMPD has risen worldwide [1]. It is important to note that when there is a combination of pTB and NTMPD, the likelihood of misdiagnosing pTB increases. Furthermore, due to similar clinical manifestations and chest X-ray signs, the differential diagnosis of these diseases may be difficult [7,8,9,10].

Given that there is a high incidence of pulmonary diseases in patients affected by bronchiectasis, cystic fibrosis, chronic obstructive pulmonary disease (COPD), and idiopathic pulmonary fibrosis, as well as the predisposition of these patients to acquire NTM respiratory infections, the necessity of considering NTMPD co-infections significantly increases [11,12]. Unfortunately, NTMP diseases are often indistinguishable from chronic lung diseases.

Deficiencies exist in conventional diagnostic methods for NTMPD. Rapid mycobacteria identification is possible with acid-fast staining (AFS), but it cannot differentiate between MTBC and NTM. The benchmark standard diagnostic technique is mycobacterial culture, but it is time-consuming and has a poor success rate because of rare bacterioexcretion among NTMPD patients [13,14].

Together, clinical, microbiological, and radiological criteria play a key role in defining NTMPD. A variety of features, such as nodularity, fibrocavitation, and dissemination, can be determined from high-resolution computed tomography (HRCT), but it cannot establish a diagnosis without mycobacteria identification. Treatment of pulmonary diseases caused by *nontuberculous mycobacteria* is particularly difficult because NTM encompasses more than 190 species and related subspecies of Mycobacterium, apart from MTBC [15]. This and the aforementioned rarely observed bacterioexcretion lead to insufficient drug susceptibility testing levels, which in turn cause problems with correct drug therapy prescription. Chronic and slow progression are typical characteristics of nontuberculous mycobacterial disease (NTMD). Because of some mimicry features, patients with NTMPD experience a delayed diagnosis [16]. Hence, differentiation between these two disorders is highly challenging, as mentioned above.

The purpose of this study is to summarize the existing methods for diagnosing diseases caused by mycobacteria and to evaluate their utility in the differential diagnosis of pulmonary tuberculosis and nontuberculous mycobacterial pulmonary disease. In addition, the study seeks to outline promising directions for the development of novel diagnostic approaches that may overcome current limitations and improve clinical decision-making.

## 2. Materials and Methods

A literature search was performed using three databases—PubMed/MEDLINE, Scopus, and Russian eLibrary (https://elibrary.ru/ accessed on 30 August 2025)—by three independent researchers up to 30 August 2025, including all terms possibly linked to two specific diseases: nontuberculous mycobacterial pulmonary disease and pulmonary tuberculosis. Leprosy was not included, since it exhibits different clinical symptoms and is usually not considered during a differential diagnosis for NTMPD and pTB. The following search string was applied: (“Nontuberculous Mycobacterial Pulmonary Disease” OR “Pulmonary Tuberculosis”) AND (“diagnostic” OR “identification”). No time filters were applied, but articles without at least an abstract in English were filtered out. Furthermore, where possible, a filter for the species “Human” was applied. Additionally, the literature that appeared in reference lists from relevant articles and conference proceedings was also considered and reviewed to ensure completeness. To refine the search results, article titles, abstracts, and indexed terms were scanned. All records were managed with Mendeley version 2.138.0, where duplicates were removed automatically and then searched for manually.

## 3. Nontuberculous Mycobacterial Pulmonary Disease and Pulmonary Tuberculosis Diagnostic Methods

### 3.1. Clinical Signs/X-Ray Diagnostics (Especially Computer Tomography) and Their Difficulties

The diagnosis of NTMPD requires a combination of clinical symptoms, radiological findings, and bacteriological cultures, along with the exclusion of other etiologies [17]. For NTM pulmonary infections, there are some systemic symptoms such as fatigue, malaise, and weight loss, but also other non-specific symptoms such as chronic cough with purulent sputum, dyspnea, and hemoptysis, which can be explained by other respiratory diseases as well as *Mycobacterium tuberculosis* infection [18,19]. There are distinct clinical features of tuberculosis and nontuberculous mycobacterial infections in patients with HIV. In such cases, clinical symptoms are often less pronounced despite more extensive sequelae, which complicates the timely identification of both TB and NTM infections [20,21].

Typical radiographic signs include nodules, fibrosis, cavitation, and bronchiectasis. However, these radiological abnormalities can occur in combination and can be further sub-classified into cavitation with fibrosis and bronchiectasis with nodules as fibrocavitary and nodular bronchiectatic, respectively [15]. When assessing radiological findings, fibrocavitary lesions present with areas of high opacity inside cavities, mostly in the upper lobes of the lungs. However, multilobar bronchiectasis can be observed in the middle and lower lobes with small nodules in a nodular bronchiectatic pattern [22]. Some studies show that, in NTM patients, thin-wall cavities possess pleural thickening and a smaller number of nodules that are larger than 10 mm without cavitation. Calcified lymphadenopathy and infiltration of parenchyma can be seen in pTB; there is no association with these signs in NTMPD. HRCT suggests bronchiolitis and cylindrical bronchiectasis with branching nodules as a “tree-in-bud” in NTMPD [23,24]. Fibrocavitary changes are typically found in elderly men with COPD [25], but another pattern, nodular bronchiectasis, predominates in postmenopausal elderly women with non-underlying pulmonary disease who suffer from skeletal deformities known as Lady Windermere syndrome [26]. According to computer tomography (CT) findings in one study [27], bronchiectasis patients without NTM infection demonstrated interstitial changes and peripheral ground-glass opacities, as did those with NTM infection. For the latter, the prevalence of bronchiectasis in the middle lobes, thickened bronchial walls, and larger cavities was prominent.

HIV-associated immunosuppression has the potential to further obscure the radiologic phenotype of NTMPD and pTB. Historical data reveal that NTMPD in the HIV-positive population often manifests atypically, demonstrating a shift away from upper-lobe predominance toward a more diffuse distribution, alongside less frequent cavitation and an increased propensity for lymphadenopathy or interstitial/miliary-type infiltrates. Consequently, radiographic features may diverge substantially from canonical descriptions. Clinicians must be vigilant for patterns including bronchiectasis, centrilobular “tree-in-bud” nodularity, thin-walled cavitary lesions, and non-specific consolidations, which can occur in any lung zone. A diagnosis should not be predicated on the expectation of classic apical cavitary disease. Given the overlap with other opportunistic lung infections, imaging findings must be interpreted in conjunction with culture, molecular microbiological, and immunological methods to establish a correct diagnosis [21,28].

Chest imaging plays a crucial role in the diagnosis and ongoing management of patients with NTMPD and pTB. In particular, computed tomography (CT) has been used to diagnose, monitor imaging changes, and evaluate the severity of these diseases. On the other hand, radiographic features can be suggestive of the type and activity of TB. However, interpreting chest CT images is challenging on account of their complexity. In countries with suboptimal health and surveillance systems, underdiagnosis and missed reporting of TB are common problems [23,24,25,26].

Artificial intelligence (AI) has gained significant attention in recent years, and many applications have been proposed in medical image recognition and interpretation. Deep learning (DL), a predominant element in the increasing application of AI, has made great progress in medical image analysis, including skin disease classification [8], diabetic retinopathy detection, and lung cancer screening by chest CT. DL algorithms can autonomously “learn” to predict features from manually classified initial data. Recent promising advances in CT-based DL systems have demonstrated the potential of AI-assisted radiological diagnostic technology. For example, Zhang et al. employed a ResNet-18-supervised network for the diagnosis and prediction of triage of patients with coronavirus disease 2019 (COVID-19) during the global pandemic based on CT images [29]. Therefore, end-to-end DL networks can potentially be designed and established by automatic and adaptive feature learning to achieve human expert-level performance for diagnosis and follow-up.

### 3.2. Observing Mycobacteria and Their Identification

#### 3.2.1. Microscopy

Because of the existence of nontuberculosis mycobacteria in the environment and also their colonization in airways, causing contaminated specimens, leading to false-positive test results, recent guidelines recommend that for the true diagnosis of NTMPD, three early morning sputa should be taken on different days [30]. Obtained sputum samples are analyzed by the Ziehl–Neelsen method and the fluorochrome procedure for determining the presence of acid-fast bacilli (AFB). Ziehl–Neelsen method sensitivity is reported to be low and variable, ranging from 20% to 80%, and specificity exceeding 90% [31]. Directly observing NTM in clinical samples through microscopy using fluorophores is comparable to MTBC. The sensitivity of fluorochrome staining is higher than that of Ziehl–Neelsen staining [32]. However, these methods cannot distinguish MTBC from NTM [33,34]. Although fluorochrome microscopy maintains high specificity (>90%) for detecting acid-fast bacilli (AFB), its sensitivity is highly variable (20–80%) and heavily dependent on bacterial load and technician expertise [34]. A high bacterial load of 5000–10,000 AFB/mL is required for detection. Microscopy, despite its well-recognized limitations in sensitivity and specificity, remains a straightforward and accessible diagnostic method for laboratories with constrained resources. Even at present, it continues to play an important role, particularly in patients presenting with a high bacillary burden.

AI-based microscopy was found to have a sensitivity, specificity, positive predictive value, negative predictive value, and diagnostic accuracy of 89.25%, 92.15%, 75.45%, 96.94%, and 91.53%, respectively. AI-based sputum microscopy has an acceptable degree of accuracy, PPV, NPV, specificity, and sensitivity [35]

Smear sensitivity is further reduced in patients with extrapulmonary TB, HIV co-infection, and those with disease due to nontuberculous mycobacteria [36]. Despite these limitations, the speed and wide availability of microscopy make it a critical initial screening tool for pTB and for making informed early decisions on patient isolation and treatment initiation [34].

In people living with HIV, especially those with advanced immunosuppression, NTMPD more frequently presents as a paucibacillary infection. As a result, direct smear microscopy (Ziehl–Neelsen or LED fluorescence microscopy) has less sensitivity (<30%) in HIV-positive or immunosuppressed patients. Similarly, this is the case in children as opposed to adults [37]. This reduced smear positivity reflects both the lower mycobacterial burden and the altered local immune response in the lungs [17,36].

#### 3.2.2. Culture

Culturing bacteria is a reference standard for the diagnosis of TB and NTMPD, but diagnostic results may only be achieved within 2–6 weeks [38]. Several biochemical tests are used to identify NTM, such as nitrate reduction, niacin accumulation, catalase estimation, the arylsulfatase test, and growth on MacConkey agar culturing media [39]. One of the most commonly performed biochemical tests is the p-nitrobenzoic acid inhibitory test (PNB). Due to NTM resistance to PNB, NTM are able to grow, while *Mycobacterium tuberculosis* growth is suppressed [40].

Both solid (Löwenstein–Jensen, Ogawa, Middlebrook 7H10/7H11) and liquid (MGIT 960, VersaTREK) media are routinely used. Liquid culture systems offer higher sensitivity (up to 90–95%) and a significantly shorter time to detection (10–14 days for many RGM, 14–28 days for SGM) compared with solid media. However, solid media remain essential for species differentiation based on colony morphology and pigmentation [37,39].

Modern studies report sensitivities of 80–95% and specificities close to 100% when culture is used as the microbiological reference standard [37]. Although challenging, mycobacterial culture is the most accurate and trusted method of detecting NTM in a respiratory specimen. The Mycobacteria Growth Indicator Tube, as an automated liquid culture, has the advantages of time savings and greater sensitivity over solid media culture, such as the Löwenstein–Jensen system [41].

Matrix-assisted laser desorption/ionization time-of-flight mass spectrometry (MALDI-TOF MS) has significantly accelerated the identification of mycobacterial species from positive cultures. In a 2023 clinical study [42] that analyzed respiratory specimens from 175 patients with suspected NTM pulmonary disease, MALDI-TOF MS demonstrated a sensitivity of 77.8% (95% CI 68.6–85.0) and specificity of 92.5% (82.8–97.2) for species-level identification, with corresponding PPV of 94.4% (86.8–97.9) and NPV of 72.1% (61.2–81.0) [42]. Based on these values, the estimated positive likelihood ratio (LR+) was approximately 10.4, while the negative likelihood ratio (LR−) was in the region of 0.24, indicating strong rule-in capability and moderate rule-out performance [42]. Newer MALDI-TOF MS platforms optimized for nucleic-acid-enhanced identification have reported even higher accuracy, with sensitivity up to 96.9% and specificity of 100% for mycobacterial species identification across clinical isolates and bronchoalveolar lavage samples [43]. A recent post-2019 meta-analysis further supports its utility, showing a pooled species-level identification accuracy of approximately 88% (95% CI 84–91) across multiple NTM species. Together, these findings highlight that MALDI-TOF MS offers a rapid (<30 min after culture) and reliable adjunct to culture-based diagnostics, significantly shortening time to species-level identification and supporting more timely clinical decision-making in NTMPD and TB diagnostics [44,45].

Conventional methods for detecting drug susceptibility of the *Mycobacterium tuberculosis complex* are complicated, time-consuming, and require considerable technical skills. Consequently, new techniques delivering a rapid diagnosis have been developed. Systems such as MB/Bact [46] and BACTEC 460 [47] are based on CO_2_ production, but others, including Mycobacteria Growth Indicator Tube, use oxygen consumption as a parameter [48]. Also, some techniques, such as particle-counting immunoassay, are able to detect bacterial proliferation in a minimal time, even for initially very low bacterial densities [49]. For microbial resistance, both phenotype and genotype should be considered. By applying next-generation sequencing, not only can we compare results with resistance detected phenotypically, but it is also possible to determine whether there is a relation between mutated genome regions and the quantity of drug resistance. The phenotypic drug susceptibility test (DST) is applied to test the ability of a given drug to inhibit bacterial growth. It can be used in both solid and liquid media directly on smear-positive samples or indirectly after culturing strains [50].

Culture-based methods remain the diagnostic cornerstone. However, HIV infection alters the distribution of mycobacterial growth. In patients with CD4 counts below approximately 50 cells/µL, disseminated disease—particularly due to *Mycobacterium avium complex* (MAC)—is more likely, and mycobacterial blood cultures become an important adjunct to respiratory sampling. In this subgroup, bloodstream infection may be detected even when sputum cultures are negative, highlighting the need for multi-site sampling [17,51].

These diagnostic characteristics underscore the importance of combining repeated respiratory cultures with blood cultures and species-level identification in individuals with advanced HIV disease [17].

#### 3.2.3. Molecular Methods

Molecular methods have become an important alternative and complementary approach to conventional microbiological techniques for mycobacterial identification and are increasingly recognized as a promising component of modern diagnostic algorithms [37]. Nucleic acid amplification tests (NAATs)—including PCR, nested PCR, reverse-transcription PCR, and loop-mediated isothermal amplification (LAMP)—employ species-specific molecular probes that enable targeted detection of members of the Mycobacterium tuberculosis complex as well as clinically relevant NTM species, such as *M. avium complex*, *M. kansasii*, and *M. gordonae* [27,37]. When present in quantities of ≥1 × 10^5^ organisms, NAATs typically achieve sensitivities and specificities approaching 100%, outperforming smear microscopy and providing results substantially faster than culture-based methods [37]. Consistent with this, a comparative investigation reported complete concordance between IS6110-targeted PCR and the BACTEC MGIT 960 culture system, supporting the integration of both assays into combined diagnostic workflows for routine clinical practice [37].

Presently, the application of molecular biological methods, such as the nucleic acid amplification test (NAAT), has significantly increased compared with traditional staining and culturing due to the achieved time savings [52]. One of these methods is the Amplified *Mycobacterium tuberculosis* Direct (MTD) test, based on the detection of ribosomal ribonucleic acid (rRNA) from *Mycobacterium tuberculosis* [53]. Despite the reliability of this test, it cannot verify the drug susceptibility of the *Mycobacterium tuberculosis* strain in a specimen [54]. On the contrary, the Xpert MTB/RIF system has the potential to specifically diagnose TB as well as detect rifampin-sensitive and resistant strains from a sputum sample [55]. Unfortunately, Xpert MTB/RIF cannot detect NTM and its rifampin sensitivity. Another molecular method is line probe assays (LiPA), which use hybridization-based probes for the detection of mycobacteria from a sample. The principle involves utilizing species-specific and genus-specific probes on nitrocellulose membrane strips [56]. The next-generation sequencing (NGS) method can be considered as a technique for accurate diagnosis of pTB as well as mutations responsible for multidrug resistance through direct use of the patient’s sputum [57,58,59,60].

In smear-positive specimens, differentiating MTBC from NTM is made faster using direct molecular detection. Recently, a quantitative multiplex PCR assay has been designed to detect both MTBC and NTM in clinical samples simultaneously because it has better specificity than Xpert MTB/RIF. According to one study from 2023 [61], this assay is specific for both *M. tuberculosis* and nontuberculous mycobacteria for two features. One is that the region that is chosen is exclusive to mycobacteria and not found in any other bacterial genus or species. The other is that by making minor alterations to the primer sequence, *M. tuberculosis complex* is able to be differentiated from NTM in a single test in patients with positive AFB in sputum samples [61]. Studies show there is a more likely coexistence of MTBC and NTM in patients with lung damage, but it is still a rare condition. The consequence may be an inappropriate diagnosis and treatment [8,9,14,15,16,17,62,63].

It is known that traditional acid-fast bacilli culture is more sensitive compared to acid-fast bacilli microscopy. Modern sputum *rpoB* gene analysis, or polymerase chain reaction (PCR), acts a little more specifically and sensitively than acid-fast bacilli culture, but culturing samples is still the gold standard test for the diagnosis of TB and NTMPD [64,65,66,67,68].

An article by Surendra et al. [69] evaluated high-performance liquid chromatography (HPLC) as a phenotypic method for identifying NTM species by analyzing their unique mycolic acid profiles. This method was used for the identification of non-tubercular mycobacterial infections in the past. These tests have now been replaced by molecular tests for NTM differentiation. These molecular tests identify only a limited number of NTM species and subspecies [69].

When evaluating diagnostic tests, positive and negative predictive values are crucial alongside test sensitivity and specificity, as they depend on disease prevalence in the tested population. A test with high specificity (>98%), like Xpert MTB/RIF, provides a high PPV even in moderate-prevalence settings, minimizing false positives. Conversely, in low-prevalence groups, the negative predictive value of a highly sensitive test becomes paramount for reliably excluding disease [70].

Table 1 characterizes the main identification methods for mycobacteria.

The performance of molecular assays in diagnosing NTMPD is significantly influenced by the patient’s immune status. In people living with HIV, particularly those with CD4 counts below approximately 200 cells/µL and most notably below 100 cells/µL, NTM infection frequently follows a paucibacillary course, which reduces the sensitivity of nucleic acid amplification tests (NAATs). Consequently, false-negative results occur more often in advanced HIV infections, even when clinical likelihood remains high. This limitation is most evident in assays optimized for detecting *Mycobacterium tuberculosis complex* (e.g., Xpert MTB/RIF), which may detect NTM only non-specifically or not at all [51,84].

Targeted PCR assays or species-specific molecular panels may improve diagnostic precision, but their yield still depends on adequate organism load in respiratory specimens. In immunocompromised patients with low CD4 counts, the reduced local immune response can impair sputum production and diminish the concentration of organisms in expectorated samples, necessitating bronchoalveolar lavage or repeated sampling for optimal diagnostic accuracy [39,51,84].

Metagenomic next-generation sequencing (mNGS) has shown superior sensitivity in settings of low bacterial burden, as it does not rely on amplification of predetermined targets. Studies published since 2020 demonstrate that mNGS significantly enhances pathogen detection in BAL fluid or tissue samples from HIV-positive individuals, including those with disseminated or atypical NTM infection. These findings support integrating mNGS into diagnostic pathways for patients with advanced HIV disease when conventional tests are inconclusive [85].

### 3.3. Histology

The hallmark of tissue infected by *Mycobacterium tuberculosis* is necrotizing granulomatous inflammation, containing lymphocytes and epithelioid histiocytes encircling a centrally necrotic zone, as well as multinucleated giant cells known as Langhans cells. Clearly, non-necrotizing granulomas can also be sometimes observed [86]. Occasionally, the possibility exists for misinterpreting necrotizing granulomas due to the involvement of vessels observed in MTBC, NTM specimens, and vasculitic disorders like Churg–Strauss syndrome. For this reason, pulmonary granulomatous diseases have always been a significant diagnostic challenge [87,88,89]. However, etiology differentiation between tissues with necrotizing and non-necrotizing granulomas is, to some extent, simplified via the AFB method since necrotizing granuloma tissues more often generate positive AFB results than non-necrotizing granulomas [90].

Both macroscopic and microscopic examinations of material from patients with NTMPD revealed similarities between different forms of NTMPD and tuberculosis [91,92]. Nevertheless, histological examination has drawn attention to distinct features of NTMPD [93].

Despite the vital role granulomas play in host defense, the cellular mechanisms underlying the formation of tuberculous granulomas remain only partially understood. Research on granulomas in human tissue is challenging due to limited access to clinical samples and the current state of imaging computer technologies [94]. Analysis of biopsy material enables the diagnosis of a combination of pTB and NTMPD in 50% of such patients [95]. Currently, there is a trend towards investigating the components of the immune response within granulomas, although studies specifically focusing on tuberculous granulomas remain scarce [96].

A study by Cuong et al. [97] was conducted among 216 specimens that were confirmed to have MTBC. Epithelioid cells were present in 83.23% of cases, which was the most common histopathological finding. Langhans giant cells accounted for 75.9%, and caseous necrosis accounted for 75.5%. In total, 83.3% of biopsy specimens in this study were found to have TB, as suggested by histopathological examination [97]. Tissue with typical histological lesions, including caseous necrosis, multinucleated giant cells, epithelioid cells, and macrophages known as granulomas, is believed to show tuberculosis or nontuberculous mycobacterial disease [98].

In an investigation conducted by Reddy et al. [99] on tuberculosis patterns in autopsies, it was found that the fibrocaseous variety of lung lesion was the most frequent. In total, 22.7% of lesions were found to be miliary, while 10.74% were of the pneumonic type [100].

Concomitant chronic pulmonary diseases, such as asthma, COPD, bronchiectasis, or active tuberculosis, remain a potential source of NTMPD. Clinically, the symptoms cannot be differentiated from NTMPD or concomitant pulmonary diseases due to overlapping symptoms [99]. Transbronchial biopsy can be used to definitively diagnose NTMPD by demonstrating pathological granulomatous inflammation, along with microbiological evaluation, but this must be coupled with an associated procedure [101].

Jarzembowski and Young [102] have found that the most prevalent and distinctive histological finding in NTM disease is necrotizing granulomatous inflammation, which is also present in TB.

Granulomas or detectable organisms may not be present in immunocompromised patients during tissue biopsy [102]. The presence of disseminated NTM and/or MTBC infection cannot be excluded if hallmark histologic findings are absent. In this case, the presence of Mycobacterium-foamy infected histiocytes, poorly formed granulomas, or no obvious inflammatory response should be considered [103]. Necrotizing granulomas have been observed in immunocompromised patients as a consequence of NTM infection [74]. Spindle cell granulomas, which are rare and contain numerous AFBs, are most commonly caused by NTM, but MTBC can also be responsible [75]. The presence of granulomas in lung tissue, regardless of AFB, correlates with positive cultures for NTMPD. In pulmonary granulomas with AFB and negative culture, PCR could be a tool for distinguishing between MTBC and NTM [104]. Thus, there is no consensus about the role of histology in the diagnosis of NTM infection, tuberculosis, and their combinations. The main findings and ideas are presented in Table 2.

Histopathological examination in people living with HIV reveals characteristic alterations in the tissue response to NTM infection. As CD4 counts decline, especially below approximately 200 cells/µL and most profoundly below 100 cells/µL, patients are less able to mount the structured cellular immune response required for well-formed granuloma development [24,51]. As a result, the classic granulomatous architecture typically associated with NTM pulmonary disease becomes poorly organized, hypocellular, and frequently non-necrotizing. In advanced immunosuppression, granulomas may appear ill-defined or entirely absent, replaced by diffuse histiocytic infiltrates with scattered mycobacteria. Caseous necrosis is uncommon in this setting, reflecting the impaired Th1-mediated immune activation needed to sustain a coordinated granulomatous reaction. These atypical patterns substantially reduce the diagnostic specificity of histology alone and highlight the need for correlation with culture-based and molecular methods in HIV-positive individuals [24,51].

### 3.4. The Future: Computer Vision

The heterogeneity of granulomas, even within a single individual, presents a significant challenge in understanding disease progression and developing targeted therapies for mycobacterial infections [2,3]. Traditional histopathological assessment, while fundamental, is inherently qualitative and limited in its capacity to objectively quantify this diversity.

Generally, studies did not carry out immunohistochemical mapping of granuloma cells; they are presented on small material and do not answer the question of significant differences in granulomatous inflammation in these diseases. To visualize the immune landscape in TB lung sections, tissues could be stained with many different immune markers: CD68 (macrophages), CD20 (B cells), CD3 (T lymphocytes), CD8 (CD3+ and CD8+ cytotoxic T cells), CD4 (CD4+ helper T cells), an activation marker T-cell CD137 (4-1BB), and programmed cell death-1 (PD-1) [2,3,110].

Recent advances in multiplexed imaging and spatial biology now provide high-dimensional datasets that capture the cellular composition, molecular features, and spatial architecture of granulomas in unprecedented detail [2,3]. When combined with computer vision (CV) and artificial intelligence (AI), these approaches allow unbiased, large-scale analysis across whole tissue sections, enabling the classification of hundreds of granulomas according to quantitative morphometric and immunological features rather than subjective categories. Such methods can also identify distinct architectural patterns, including the “histopathological superstructure” described by Sawyer et al. (2023) [110], which consists of an organized necrotic lipid core, an inner layer of CD68^+^ macrophages and foam cells, and an outer ring of lymphocytes and fibroblasts. CV algorithms trained on digitized whole-slide images can automatically segment these zones, assess whether the overall structure is preserved or disrupted, and link such descriptors to clinical outcomes [110].

The integration of high-plex technologies such as Imaging Mass Cytometry (IMC) and CO-Detection by indEXing (CODEX) further expands this analytical power. As shown by McCaffrey et al. (2022) [111], more than thirty proteins can be simultaneously mapped, providing insights not only into cellular identity but also into functional and activation states within the granuloma microenvironment. With CV, these multiplex datasets can be mined to identify and enumerate key populations—such as IFN-γ^+^ CD4^+^ T cells or PD-1^+^ exhausted T cells—and to perform neighborhood analyses that reveal statistically significant patterns of cellular interaction. In turn, these analyses highlight protective immune responses associated with specific spatial arrangements while also identifying ineffective responses characterized by disrupted crosstalk or immunosuppressive niches. Detecting such “defective” spatial immune signatures in patient samples may point to targets for immunomodulatory interventions aimed at restoring effective host defense [110,111,112].

Ultimately, algorithms trained on multiplexed datasets can integrate information on morphology, molecular expression, and spatial context to generate predictive models of disease progression, relapse risk, or therapeutic response, all directly from biopsy material. By combining traditional pathology with AI-driven computational approaches, it becomes possible to uncover cellular interactomes and signaling pathways that underlie granuloma formation and containment, opening new opportunities for rational therapeutic targeting [2,3,109,110,111,112].

### 3.5. Immunological Tests

The diagnosis of *Mycobacterium avium* complex (MAC) disease is possible with a commercial kit that measures anti-glycopeptide lipid immunoglobulin A. Serology tests are still not widely used for diagnosing NTMPD [79].

There is a type of immunological test examining the presence of IgG antibodies to mycobacterial antigen A60 for the detection of *M. abscessus (MAC)* pulmonary disease [81]. The A60 antibody titers have been characterized as having both sensitivity and specificity for *M. abscessus* pulmonary disease. However, these antibodies can also arise in tuberculosis [113]. Thus, more research is needed in this field for the proper diagnosis of NTMP diseases. Interferon gamma release assays are not used for the diagnosis of NTM due to the possible generation of false positives. Less interferon gamma release is observed in patients with MAC pulmonary disease in response to MAC antigens compared to individuals without disease having a positive skin test response to MAC sensitizer [76].

The urinary lipoarabinomannan test (LAM test) is used for the detection of LAM, a part of the cell wall of MTBC, which is released by metabolically active or lysing bacterial cells. This test is not highly sensitive to nontuberculous mycobacteria due to its specificity to tuberculous-like mycobacterial infections and low cross-reactivity. The presence of significant concentrations of LAM in urine can indicate a more likely infection with the *M. tuberculosis complex* [81].

### 3.6. Immunohistochemistry (IHC)

Immunohistochemistry (IHC) has the ability to confirm the involvement of granulomatous tissue and demonstrate the immunolocalization of MTBC antigens.

According to one study by Karimi et al. [90], 23 confirmed TB samples containing enough granuloma tissues were analyzed using Ziehl–Neelsen staining and IHC staining with pAbBCG. While Ziehl–Neelsen staining was positive in only 9 cases, IHC staining showed positive results in all 23 samples. The cytoplasmic and round fragmented bacilli were stained in a positive manner by IHC. The periphery of the granuloma had significantly more positive staining attributed to epithelioid cells than the center of it, as indicated by this study. Positive staining could also be seen in plasma cells, endothelial cells, macrophages, and fibroblasts. Thus, the IHC technique can be utilized to evaluate the presence of mycobacterial antigens and tissue structure [90].

There are immunohistochemical (IHC) markers that may hold potential for use in the diagnosis of NTMD and tuberculosis, and they are unequivocally implicated in the pathogenesis of these diseases (Table 3).

## 4. Conclusions

It is important to identify and promptly diagnose co-infection of pTB and NTMPD, since each one in turn contributes to pathogenesis. Thus, it is often impossible to achieve complete patient recovery by treating pTB or NTMPD in isolation when both are in combination.

NTMP diseases are one of the main causes of opportunistic diseases, such as pulmonary diseases, especially in HIV patients or immunosuppressed patients, such as those with TB. Currently, there are no unique diagnostic methods for pTB and NTMPD co-infection. Some diagnostic techniques have been developed for pTB and NTMPD, but there are some possible limitations due to similar symptoms but different therapeutic regimens. Thus, tools that can simultaneously diagnose TB and NTMP are urgently needed. Not only are accurate diagnosis and appropriate treatment principal goals, but the prevention of antibiotic resistance must also be integrated into any public health plan.

Studies of granulomas in human tissue are frequently hindered by restricted access to clinical samples and technological constraints in imaging. Furthermore, the strains of laboratory mice commonly employed in research fail to entirely replicate the distinctive attributes of human granulomas. These challenges are compounded by difficulties in granuloma classification arising from the diverse sizes and histopathological characteristics of affected human tissues.

## 5. Perspectives

Many investigations tend to focus on characterizing specific types of lesions in isolation, often delineating between necrotizing and non-necrotizing forms. To fully comprehend the variations in cellular composition and organization of tuberculous lesions in the human lung, the utilization of contemporary methodologies, such as immunostaining and computer image analysis, could be imperative [69,80,93,94,117].

Despite the availability of microbiological, radiological, and clinical methods for diagnosing mycobacterial diseases, significant challenges remain in differentiating them from tuberculosis. Emerging approaches based on computer vision and artificial intelligence hold considerable promise in this context, particularly in the analysis of images, morphology, histology, and immunohistochemistry. Subtle variations in cell density, heterogeneity, spatial distribution, and intercellular interactions within granulomas often escape reliable human assessment, yet become detectable through digital microscopy combined with segmentation algorithms and neural network-based image analysis. These technologies enable the identification of hidden patterns and provide robust quantitative metrics, offering a new pathway toward a more accurate and reproducible understanding of pathogenesis and differential diagnosis.

## 6. Limitations of the Study

This review has several limitations. The work is a narrative, not a systematic review. Study selection criteria may not have encompassed all potential information sources. The human subjective element in choosing literature could potentially result in valid sources being discarded during the initial filtering stages. Consequently, the review may be incomplete, and the process may not be fully reproducible.

Although modern diagnostic methods for distinguishing NTMPD from pulmonary tuberculosis are well described in the contemporary literature, there is a lack of widely accessible studies that clearly evaluate their clinical value and real-world performance. In addition, in order to examine and evaluate some aspects of diagnosis, particularly historical criteria and early laboratory approaches, available publications are older because recent comprehensive studies are limited. These factors may influence the depth of the analysis and highlight the need for well-designed prospective research into assessing the practical impact of current diagnostic tools.

## Figures and Tables

**Table 1:** Comparative Characteristics of Diagnostic Methods for MTB and NTM.

Type of Diagnostic Methods	Diagnostic Method/Key Characteristics	Sensitivity	Specificity	Turnaround Time	Accessibility/Cost	Notes	References
Microscopy	Acid-fast staining (AFS) Ziehl–Neelsen	Low–moderate 20–75%	<90%	Hours to 3 days (very fast)	High	Cannot differentiate between MTBC and NTM.	[32,33,34,37,71,72]
	Acid-fast staining (AFS) fluorochrome	60–80%	exceeding 90%		Intermediate		
Culture	Mycobacterial culture (solid media, e.g., Löwenstein–Jensen)	80–95% High * (gold standard)	close to 100% (gold standard)	30–60 days (slow)	High, but time-consuming.	Cheap. Poor success rate for finding NTM due to rare bacterioexcretion	[36,37,38,41,47]
	Mycobacterial culture (liquid media, e.g., MGIT)	95–100% *	Close to 100%	15–30 days	Intermediate. Availability depends on state-of-the-art laboratory equipment	Intermediate. Reference standard. Used for subsequent drug susceptibility test (DST).	
Culture (phenotypic identification)	Biochemical tests (e.g., PNB inhibition)	High * for MTB	High * for differentiation	Weeks (after culture growth)	High. Specialized laboratory	Relies on prior culture growth. NTM resistance to PNB. MTBC is inhibited by PNB, while NTM grows.	[37,38,39]
	High-performance liquid chromatography (HPLC) analysis of the number of carbon atoms in mycolic acid found in the cell walls of NTM species.	Variable Varies by species: *M. abscessus* complex—100% *M. fortuitum complex*—95.2% *M. avium complex*—97.1% Lower * for rare species	Varies by species and database. Very high * for common species	Weeks	Low. Requires specialized equipment	This method identifies slowly growing NTM species such as MAC and *M. kansasii*, but it is less specific in identifying rapidly growing mycobacteria (RGM) accurately	[37,69]
	MALDI-TOF Mass Spectrometry	High (for identification from culture)	Very high (>95% for common species)	Minutes to hours (after culture growth)	Low. Requires specialized equipment and an updated database	Revolutionizes workflow by providing rapid, accurate species identification from positive cultures. Performance is database-dependent	[37,44,73]
Molecular	Nucleic Acid Amplification Test (NAAT)—General (e.g., MTD test)	High for MTBC 90–95%	High for MTB >95%	1–2 days	Intermediate	The MTD test is specific for MTB and does not detect NTM.	[37,52,53]
	Xpert MTB/RIF Assay	High * for MTB	Up to 99%. Very high for MTB	<2 h	Low	Specifically diagnoses MTBC and detects rifampicin resistance. Cannot detect NTM.	[37,57]
	Line Probe Assays (LiPA)	High *	Up to 99%. Very high *	1–4 days	Low	Can differentiate between the MTB complex and various NTM species.	[37,57]
	Quantitative Multiplex PCR (Newer assays)	High *	Up to 99%. Very high *	1–4 days	Low	Some newer assays can simultaneously detect and differentiate MTBC and NTM in a single test.	[37,61]
	Next-Generation Sequencing (NGS)	Very High 100%	Up to 99%. Very High *	Days to weeks	Low	Can identify all species and detect resistance mutations directly from sputum.	[37,57,58,59,60]
Microscopy	Histology and Microscopy (Granuloma assessment)	Low * for organism detection	Low * for etiology	Days	Intermediate–High	Necrotizing granulomas suggest mycobacterial disease but cannot reliably distinguish MTBC from NTM.	[37,74,75,76,77]
	Immunohistochemistry (IHC)	Very High (for antigen detection) 100%	High *	Days	Low	Can detect mycobacterial antigens in tissue with higher sensitivity than AFS but may not differentiate MTBC from NTM.	[37,77,78]
Immuno- diagnostic	Serological Tests (e.g., Anti-GPL, A60 antibodies)	Variable, low to moderate Up to 87%	Variable, low to moderate Up to 95%	Days	Low. Primarily for research use	Not recommended for routine diagnosis due to cross-reactivity (e.g., with TB) and lack of standardization.	[37,79,80]
	Interferon Gamma Release Assays (IGRAs)	High * for MTB infection	High * for MTB infection	Days	Intermediate. Widely available	Not used for NTM diagnosis due to cross-reactivity and high false-positive rates.	[81,82]
	Urinary Lipoarabinomannan (LAM) Test	Low * to moderate for MTB	High * for mycobacteria	Minutes	Intermediate. Point-of-care	Highly specific for MTBC; very low cross-reactivity with NTM.	[83]

* Numeric values are not specified.

**Table 2:** TB and NTM granuloma comparison.

Source	Object of Study	Methods	Characteristics of NTM Granulomas	Characteristics of TB Granulomas	Conclusions
Jing Jing Li et al. [105]	Skin biopsies (NTM infection) from 13 patients	Histology (H&E, AFB) (PCR) on the paraffin blocks	In immunocompetent patients: pseudoepitheliomatous epidermal hyperplasia, intraepithelial abscesses, transepidermal elimination, and dermal granulomatous inflammation with necrosis and suppuration. In immunocompromised patients: suppurative inflammation with little granuloma formation and numerous acid-fast bacilli.	N/A	According to this study, paraffin block PCR (positive in 4 of 13 cases, 31%) is not superior to conventional culture (positive in 11 of 13 cases, 85%) in detecting cutaneous NTM infection.
R. Bartralot et al. [106]	Skin biopsies (NTM infection) from 27 patients	Histology	Epidermal changes like acanthosis and pseudoepitheliomatous hyperplasia were primarily associated with *M. marinum*. In immunosuppressed patients, the infiltrate was deeper, involving subcutaneous tissue (100%) with frequent abscesses. A marked granulomatous reaction was seen in 83% of immunocompetent versus 60% of immunosuppressed patients, with chronicity correlating to granuloma formation in the latter. A foamy histiocytic infiltrate was noted in three AIDS patients, and panniculitis was found in 8 biopsies.	N/A	Suppurative granulomas are the most characteristic feature in skin biopsy specimens from cutaneous NTM infections. Some histopathological patterns seem more prevalent in immunosuppressed patients.
D. Jain et al. [77]	Review of acid-fast bacteria (AFB) detection tips	Histology	Necrotizing granulomas. Pink (“caseating”) necrosis. Not specific to tuberculosis.	Lung biopsies are seldom indicated unless non-invasive modalities fail to provide a diagnosis, or the clinical setting is atypical, or if rapid diagnosis is essential. Lung biopsies are commonly performed in the evaluation of individuals with lung nodules or masses, since such lesions often raise the possibility of other granulomatous infections or lung cancer. Necrotizing granulomas are the most common benign finding in core needle biopsies, and mycobacteria can be demonstrated in a subset of these cases.	M. tuberculosis and NTM cannot be reliably distinguished by tissue reaction or bacterial morphology on acid-fast stains. The presence of AFB in necrotizing granulomas does not confirm tuberculosis, as NTM can present similarly. Morphological differences were mostly observed in immunocompromised patients with NTM and were not directly compared to classical tuberculosis. The presence of mycobacteria within spindle cell pseudotumors or macrophage sheets is more suggestive of NTM/MAC, but the practical utility of this feature remains questionable.
N. Mehta et al. [107]	Brief overview of skin atypical mycobacterial infections	Histology	Histopathology typically reveals a mixed suppurative and granulomatous reaction, which is the most common pattern (80%). Two less common variants are a predominantly suppurative form with neutrophilic abscesses and no granulomas (15%), and a predominantly granulomatous form with minimal neutrophils, with or without caseous necrosis (4%).	N/A	The histopathology of NTM infections is not specific and cannot reliably differentiate them from other causes. Although the yield of special staining varies significantly, a biopsy is required in all suspected NTM cases.
M. Kraus et al. [108]	Computed tomographic (CT) studies and clinical courses of eleven patients with laboratory-confirmed diagnosis of NTM	Radiology, overview	Microabscess. Several histopathological features, including predominantly noncaseating granulomas, ill-defined granulomas, the presence of microabscesses, and a relatively small number of giant cells, support the clinical diagnosis of NTM infection.	Caseating granuloma Well-defined granuloma Numerous giant cells	Cervical lymphadenitis with classic NTM symptoms likely stems from atypical mycobacteria. Key histological indicators include noncaseating and ill-defined granulomas, microabscesses, and a minimal presence of giant cells.
M. Samsonova and A. Chernyaev [109]	lungs	Histology, overview	Granulomas in NTM are more variable and may include microabscesses.	Classical TB granulomas	The author believes that TB and NTM granulomas are indistinguishable.
L. Lepekha et al. [93]	Operating material from 69 patients (lungs, lymph nodes)	Histology, partially immunohistochemistry	Infections caused by *M. avium* and *M. intracellulare* feature extensive lymphocytic infiltrates and non-necrotic, histiocytic macrophage granulomas. These granulomas often merge into larger aggregates with diffuse fibrosis. In the lung, dense peribronchial lymphocytic infiltrates and granulomas can compress airways, causing lumen narrowing and epithelial metaplasia. A key change in terminal acinar regions, especially with fast-growing mycobacteria, is chronic constrictive bronchiolitis, characterized by epithelial necrosis, luminal mucus, and lymphocytic infiltration of the walls.	Characteristic morphological features of TB, with classical TB granulomas, reflecting high, moderate, or low activity of the inflammatory process, have been identified. The development is accompanied by fullness, impairment of permeability of the circulatory stream, and the presence of vascular fistulas, the detection of which varies depending on the activity of tuberculosis.	NTMD develops against a background of tuberculous inflammation and in areas unaffected by tuberculosis foci. The histological features differ between slow-growing and fast-growing species, characterized by productive changes in the former and more pronounced, destructive changes in the latter.

N/A—not available.

**Table 3:** Summary of studies: immunohistochemical marker expression in tuberculosis versus nontuberculous mycobacterial infections.

Sources	Marker	Context of Use	Behavior in TB	Data/Assumptions for NTM	Comments/Limitations
[50,75,77,82,86,90,94,105,106]	CD68	General macrophage marker to identify the primary cellular component of granulomas.	Presumed to be highly expressed in granuloma macrophages.	Presumed to be expressed in granuloma macrophages.	A ubiquitous marker for histiocytic cells; likely not useful for differentiating TB from NTM but essential for confirming granulomatous inflammation.
[90,94,105]	CD20	Marker for B lymphocytes; used to assess B-cell presence and potential tertiary lymphoid structure formation within granulomas.	N/A	N/A	The provided data focuses on T-cell and cytokine markers.
[77,81,82,90,94,102,105]	CD3	Pan-T-cell marker to identify total T-cell infiltrate within granulomatous tissue.	Presumed to be highly expressed, indicating a significant T-cell presence.	Presumed to be expressed.	Like CD68, it is a general marker for the adaptive immune response but lacks specificity for distinguishing the type of mycobacterial infection.
[77,81,82,90,94,110,114,115]	CD4	Identifies T-helper cells, crucial for granuloma formation and macrophage activation via IFN-γ.	Described as essential for immune control. A specific subset re-wires granuloma networks upon reinfection. They are a major source of IFN-γ.	Assumed to be present, but the specific spatial organization and functional state (e.g., expression of CXCR3) may differ from TB.	Critical for protective immunity. Their functional state (e.g., cytokine production and expression of inhibitory markers) is more informative than mere presence.
[77,80,81,90,94,110]	CD8	Identifies cytotoxic T cells; can contribute to killing infected cells.	Present in granulomas, but their specific role is less defined compared to CD4+ T cells	Not specifically mentioned for NTM.	There is no clear comparative analysis of CD8+ T cells between TB and NTM.
[12,50,81,82,111,116]	TNF-α	Pro-inflammatory cytokine critical for maintaining granuloma structure and containing infection.	Essential for granuloma integrity. The amount and location of TNF are crucial; too much can be associated with worse outcomes. A few T cells in the granuloma produce it.	Assumed to be involved in granuloma maintenance.	Its role is complex; absolute levels are less important than its localized and controlled production within the granuloma.
[78]	TNF-beta	Cytokine analyzed as a potential discriminatory IHC marker.	Lower expression in tuberculous granulomas compared to nontuberculous granulomas.	Higher expression in nontuberculous granulomas compared to TB.	While TNF-beta (lymphotoxin-alpha) is conventionally recognized as a pro-inflammatory cytokine, whose upregulation would be expected in chronic inflammatory conditions like tuberculosis, findings from some studies present a counterintuitive picture. Specifically, the immunohistochemical analysis by Seo et al. [98] reveals a paradoxical decrease in TNF-beta expression within tuberculous granulomas compared to their nontuberculous counterparts. This inverse relationship underscores its potential role as a valuable discriminatory marker in the differential diagnosis of TB and NTM lymphadenitis, particularly when integrated into a multi-marker panel.
[115]	CD137	Marker of T-cell activation.	Expressed on a high frequency of MTB-specific CD4+ T cells within granulomas, indicating recent antigen recognition and activation.	N/A	A promising marker for detecting actively engaged T cells in TB, but its utility for TB vs. NTM differentiation is unknown.
[110,114]	PD-1	Inhibitory receptor marking T-cell exhaustion; target for checkpoint blockade.	Highly expressed on antigen-specific CD4+ T cells during active TB. The granuloma core is enriched for its ligand, PD-L1.	N/A	High expression in TB suggests T-cell functional impairment. Blockade may improve outcomes but risks immunopathology.
[78,105,106]	LACT (Lactoferrin)	Iron-binding protein involved in host defense; investigated as a discriminatory IHC marker.	Higher expression in tuberculous granulomas. Shows high specificity (100%) but relatively weak staining.	Lower expression in nontuberculous granulomas.	Despite high specificity, its weak and variable expression limits its use as a standalone test. It is a key component of the best combined marker score.
[78,114]	IDO	Immunosuppressive enzyme that modulates T-cell responses.	Higher expression in tuberculous granulomas. Plays an immunosuppressive role, and its blockade may reorganize the granuloma to improve bacterial control.	Lower expression in nontuberculous granulomas.	Has a dual role as both a useful diagnostic marker and a potential immunotherapeutic target. Part of the effective combined marker score.

N/A—not available.

## Data Availability

Data sharing is not applicable to this article.
